# Sperm morphology, sperm motility and paternity success in the bluethroat (*Luscinia svecica*)

**DOI:** 10.1371/journal.pone.0192644

**Published:** 2018-03-06

**Authors:** Camilla Lo Cascio Sætre, Arild Johnsen, Even Stensrud, Emily R. A. Cramer

**Affiliations:** Natural History Museum, University of Oslo, Oslo, Norway; National Cancer Institute, UNITED STATES

## Abstract

Postcopulatory sexual selection may select for male primary sexual characteristics like sperm morphology and sperm motility, through sperm competition or cryptic female choice. However, how such characteristics influence male fertilization success remains poorly understood. In this study, we investigate possible correlations between sperm characteristics and paternity success in the socially monogamous bluethroat (*Luscinia svecica svecica*), predicting that sperm length and sperm swimming speed is positively correlated with paternity success. In total, 25% (15/61) of broods contained extra-pair offspring and 10% (33/315) of the offspring were sired by extra-pair males. Paternity success did not correlate significantly with sperm morphology or any aspects of sperm motility. Furthermore, sperm morphology and sperm motility did not correlate significantly with male morphological characters that previously have been shown to be associated with paternity success. Thus, the sperm characteristics investigated here do not appear to be strong predictors of paternity success in bluethroats.

## Introduction

In species where females copulate with two or more males, postcopulatory sexual selection may take place in the form of sperm competition [1] or cryptic female choice [2]. In sperm competition, sperm from two or more males compete to fertilize a set of ova, and the outcome may depend on certain qualities of the sperm cells and sperm producing tissues, such as sperm length, sperm swimming speed or sperm numbers [3]. Cryptic female choice is the ability of females to control which male fertilizes their eggs after having copulated with several males [2]. Mechanisms of cryptic female choice include, for example, active ejection of less preferred sperm by the female [4] and differential chemical attraction between the sperm and egg, depending on the compatibility of their genotypes [5,6]. Cryptic female choice may counteract the effects of sperm competition, unless certain sperm traits are related to male qualities preferred by females [7,8]. While comparative studies give clear evidence that sperm traits differ among taxa according to the opportunity for postcopulatory sexual selection (insects: [9], mammals: [10], birds: [11–13], relatively few studies have been conducted within species, and particularly in wild animals, to understand how this evolutionary pattern arises.

Intuitively, in a sperm competition situation, a faster swimming sperm cell should have higher fertilization probability since it on average would reach the egg (or, for species with sperm storage, female sperm storage organs) before a slower sperm cell. Sperm swimming speed, as well as the proportion of motile sperm in ejaculates, has been shown to correlate with levels of promiscuity between species [12,14]. In intraspecific experiments controlling for sperm quantity, faster swimming sperm have repeatedly been shown to have higher fertilization success in a variety of taxa [15–22]. Studies on wild birds are scarce, but sperm swimming speed may not have a similarly strong effect on fertilization success in natural settings when among-male variation in sperm quantity [23] or timing of copulations [24] may override the effects of sperm swimming speed. Sperm storage by the females in specialized sperm storage tubules (SSTs) may also dissociate initial sperm swimming speed from fertilization. Swimming speed may be important in gaining access to the SSTs, but the longevity of the sperm may determine which sperm eventually gains access to the eggs [25].

Sperm morphology may affect fertilization success through various effects on swimming speed, as well as through direct effects. For example, a longer flagellum may propel the cell faster [10,26] and a longer midpiece may contain a larger mitochondrion that provides more energy [23,27]. The shape and length of the head may also be important, since the head produces drag, which counteracts the propulsion of the flagellum [27–29]. Between-male variation in sperm length has been found to decrease with rate of extra-pair paternity among species [30–32]. As sperm competition increases, stabilizing selection on sperm morphology may decrease the variation between males, and sperm outside of the optimal range may be selected against. Such direct selection on sperm morphology could occur, for example, via interactions with the female’s SSTs [33].

Since females of many passerine species copulate with multiple males [34], and there is evidence for selection on sperm morphology across passerine species (e.g. [12,13,32]), sperm traits may have significant effects on male reproductive success in this group of birds. Selection on sperm morphological traits may be stronger than selection on sperm numbers, as extra-pair paternity rates correlate more tightly with variability in sperm length (a negative correlation) than with relative testes mass (a proxy for sperm numbers) [32]. However, the few studies conducted on wild populations thus far have given mixed results. Laskemoen et al. [23] found some evidence that midpiece length may indirectly affect fertilization success in a nestbox population of tree swallows (*Tachycineta bicolor*), but sperm quantity seemed to be more important. In a free-living population of superb fairy-wren (*Malurus cyaneus*), sperm with a longer flagellum and a relatively smaller head secured more within-pair fertilizations, whereas sperm with the opposite morphology was more successful in obtaining fertilizations in other nests [35]. Cramer et al. [36] did not find any significant association between fertilization success and sperm morphology in a nestbox population of house wrens (*Troglodytes aedon*), nor did Edme et al. [37] in collared flycatchers (*Ficedula albicollis*). Since only four studies have examined selection on sperm morphology in wild passerine populations, and only one study has examined selection on sperm velocity [23], more work is critically needed to understand the within-population dynamics that underlie the interspecific patterns detected in passerines.

In this study, we investigate how sperm characteristics relate to male reproductive success in a Norwegian population of bluethroats (*Luscinia svecica svecica*). The bluethroat is a small passerine bird with medium to high levels of extra-pair paternity (7–33% of young and 8–76% of broods, variation depending on year [38]). Available evidence suggest that sperm competition could play an important role in determining patterns of paternity in this species, as success in extra-pair fertilizations is only weakly related to male coloration and more strongly related to male age [39], which is corroborated by larger testes and seminal glomera of older males [40]. On the other hand, cryptic female choice has also been suggested to occur, since extra-pair offspring have a higher cell-mediated immune response and higher heterozygosity than their within-pair half-siblings, implying that extra-pair mates have a higher genetic compatibility [41,42]. Irrespective of mechanism, strong selection on sperm morphology may have occurred in the recent past in this species, since sperm length varies dramatically among recently diverged bluethroat subspecies (from 200.6 to 225.2 μm [43]).

Our main aims are two-fold: First, we test the hypothesis that sperm characters are related to fertilization success. We focus on sperm swimming speed, on morphological traits thought to underlie swimming speed variation (i.e. the relative length of the midpiece and the ratio of the flagellum to the head), and on morphological traits potentially under selection (i.e. total sperm length). We predict that paternity success should be positively associated with sperm length and/or sperm swimming speed (e.g. [10,15]) if directional selection is operating in the population. Alternatively, we may expect a non-linear relationship between sperm length and paternity success if sperm size is under stabilizing selection [32]. Second, we test whether sperm characters are related to male traits that have been shown to co-vary with fertilization success, specifically the width of the red border (a prominent feature of the male ornamental throat patch [39]) and age. Correlations between characteristics of the male and sperm characteristics have been observed in birds (e.g. [44,45]) and could have important implications for trait evolution [46]. Our goal was first to assess correlations among traits, and second to account for the potential impact of correlated pre-copulatory traits on the relationships between sperm traits and fertilization success [47].

## Materials and methods

Fieldwork was conducted in the valley of Øvre Heimdalen, Øystre Slidre, in Oppland, Norway (61°25’N, 8°52’E) during spring/summer in 2013, 2014 and 2015. We caught adult bluethroats (*N* = 187) with mist nets. All adults were banded and bled by puncturing the brachial vein. We measured the length of the tarsus (between the extreme bending points [48]) to the nearest 0.1 mm with a slide caliper, the length of the wing (flattened and straightened [49]) to the nearest 1 mm with a wing ruler, and body mass (to the nearest 0.5 g) with a Pesola 50 g spring balance. For males, we measured the width of the red border of the throat patch, a trait that may be subject to female mate preferences and affect which males obtain copulations [39]. The age of the bird was determined as either second year (2k) or older (3k+) by inspecting the coverts of the wings [49].

Chicks were weighed at least two days after hatching and bled by puncturing the femoral vein. Unhatched eggs were collected (*N* chicks and unhatched eggs combined = 377). All applicable international, national, and/or institutional guidelines for the care and use of animals were followed. All birds were released immediately upon completing sampling. Ethical permissions for fieldwork were to AJ (license 2014/53673) from the Norwegian Animal Research Authority.

In total, 145 ejaculates, from 105 males, were obtained by gently massaging the cloacal protuberance, as described in Wolfson [50]. Within the same year, most of the repeated samples were collected on the same day. Some were separated by a few days, while the maximum was 22 days. The ejaculates were diluted in a microcentrifuge tube containing phosphate buffered saline (PBS) preheated to 40°C. Sperm motility (i.e., swimming speed and the proportion of motile sperm, see below) was recorded immediately upon collection and the remaining sperm was fixed in 5% formalin for later morphometry measures. We used PBS in taking measurements of sperm motility because measurements in PBS are correlated with measurements in a medium derived from blood plasma in bluethroats [40], suggesting that measurements taken in PBS are representative of measurements in more biologically relevant, but more logistically challenging, media.

Some of the ejaculate samples (*N* = 41) were used in experiments for an unrelated study (Cramer et al. 2016 [51]), so there was some variation in how the sperm recordings were taken. For a subset of the experimental recordings (*N* = 23), ejaculates were put into 12 μl of PBS, and 2 μl of this “stock” suspension was put into 5 μl of female fluid two times and 5 μl of PBS one time as a control (i.e. three times per ejaculate); from these experiments, we included only data from the control treatment (see Cramer et al. [51] for details). In other experiments aimed at understanding dilution effects on sperm motility (*N* = 18), we diluted the stock suspension in a ratio of 2 μl of stock suspension to 5 μl of PBS (*N* = 18), and we filmed the stock and diluted suspensions in multiple slide chambers. Here, to obtain higher sample sizes, we averaged sperm motility parameters across the dilute chambers per ejaculate. For the non-experimental recordings, ejaculates were simply diluted into 20–40 μl of PBS, depending on the density of sperm cells obtained. For all non-experimental samples (*N* = 99), 3 μl of diluted sperm was placed in a preheated microscope slide (depth 20 mm; Leja Products BV, Nieuw-Vennep, the Netherlands). Excluding experimental males did not alter any of our results qualitatively. Each slide was mounted on a stage warmer maintained at a constant temperature of 40°C (2013: MiniTherm stage warmer, Hamilton Thorne Biosciences, Beverly, MA; 2014 and 2015: Tokai Hit TP-S heated microscope stage, which allowed us to observe and film a greater proportion of the slide area, Tokai Hit Co, Fujinomiya-shi, Shizuoka-ken, Japan). Sperm movement was recorded through a phase contrast microscope (CX41, Olympus, Japan) with a digital video camera (HDR-HC1C, Sony, Tokyo, Japan). Each sperm sample was recorded in different locations across the slide chamber to reduce the probability of tracking the same cell twice.

### Sperm morphology

Digital pictures were taken with a Leica DFC420 camera mounted on a Leica DM6000 B digital light microscope at 160 x magnification, and the images were processed in Leica Application suite version 4.1. Sperm cells consist of three components: head, midpiece and tail (i.e. exposed flagellum). The lengths of these components were measured separately, and a number of variables were calculated based on these measurements, including total sperm length (head + midpiece + tail), flagellum length (midpiece + tail), F:H ratio (flagellum/head), and M:TSL (midpiece/total sperm length). All sperm measurements were performed blindly with respect to male identity, by one measurer (ES).

At least 10 cells for each of 104 males were measured for sperm morphology (35 in 2013, 24 in 2014, 35 in 2015, and an additional 10 males that were measured in two or more years). Measuring 10 cells gives unbiased values for total sperm length [52]. To gain additional power to detect selection on variation in sperm morphology, and for the purpose of another study [53], we measured an additional 20 cells per male (for a total of 30 cells per male) for the 69 males captured in 2013 and 2014. The lengths of the sperm components were averaged, and the F:H ratio and M:TSL were calculated separately for each cell and then averaged. We tested whether these measures correlate significantly with sperm swimming speed, but this was not the case (F:H ratio: *t* = -0.58, *p* = 0.56, M:TSL: *t* = -1.78, *p* = 0.08). Note that morphology was not assessed for all ejaculates where velocity was recorded (for example, if two ejaculates were collected during a single capture, we often recorded only velocity), and some ejaculates had too few cells to assess velocity, but measuring morphology was possible. For one male, only a velocity measure (not morphology) was taken.

### Sperm motility

Sperm swimming speed and the proportion of motile cells was measured with computer-assisted sperm analysis (HTM-CEROS II Sperm Analyzer; Hamilton Thorne Research, Beverly, MA), as described in Kleven et al. [12]. The sperm analyzer was set at a frame rate of 50 Hz for 25 frames (i.e. sperm cells were tracked for 0.5 seconds). As an estimate of sperm swimming speed, we used the curvilinear velocity (VCL), which is the velocity of the point-to-point sperm track [23]. The computer program also calculates the average path velocity (VAP) and the straight line velocity (VSL), which we used to exclude suboptimal sperm tracks (see below).

The number of static and motile cells and the proportion motile cells were also calculated. Filters were applied to exclude inaccurate tracks and incorrect detections for all the measurements in all three years, except for proportion motile sperm in 2013 (see below). In order to qualify as good motile tracks, and contribute to the mean sperm velocity, sperm tracks had to have at least 10 detection points, zero gaps in the detection series, linearity (= (VSL/VCL)*100) of 60 or greater, straightness (= (VSL/VAP)*100) of 80 or greater, and elongation (ratio of sperm head width to head length) of 50 or less. Also, no single movement could be more than five interquartile ranges greater than the median length of movements for that sperm track. Moving cells with VAP under 50 or VSL under 25 were considered static (they were likely moving because of drift or software analysis issues). We set a cutoff of 10 good motile tracks per male, and excluded all males with sperm velocity measurements under this value (*N* = 9). One exception was made when testing the repeatability of sperm velocity between the years. To avoid losing multiple data points, we lowered the cutoff to 5 good motile tracks in this analysis.

In the estimates of proportion motile cells, different settings were used in 2013 compared to the other two years because of different video quality, due in turn to improved equipment that allowed us to collect data on more cells. For 2013 the number of motile tracks (including motile tracks that fail the above filters) was divided by total number of sperm cells. For 2014 and 2015, an elongation filter was applied, so moving points with elongation over 50 was eliminated from the dataset. We set a limit of 30 cells in total for calculating proportion motile cells, and excluded males with measurements under this value (*N* = 3).

We also calculated the number of sperm cells per microliter for each recording, based on the number of detected cells and the total volume of the microscope slide filmed. This measure is unlikely to be a reliable proxy for sperm quantity in a natural ejaculate, as it only reflects the density of sperm in the recording after having been diluted. However, we assessed whether sperm density during recording correlate with VCL (which would necessitate accounting for it in analyses of sperm velocity and paternity success). This was not the case (*t* = -1.4, *p* = 0.17), and thus, we did not include this parameter as a covariate in our models relating VCL to other variables.

### Parentage analysis

DNA was extracted from blood samples using an E-Z 96 Blood DNA Kit (Omega Bio-Tek (D1199-01)) or DNeasy Blood & Tissue Kit (Qiagen), following the manufacturers’ protocol. From the tissue samples of unhatched eggs, DNA was extracted with an E.Z.N.A. ® Tissue DNA Kit (Omega Bio-Tek).

In 2013 and 2014, 22 microsatellite markers (S1 Table) were amplified using polymerase chain reaction (PCR) (GeneAmp® PCR System 9700 (Applied Biosystems)) in 5 multiplexed panels. In 2015, due to logistical constraints, only eight of these markers were amplified, in two multiplex panels. The PCRs were run in 10 μl volume (per sample) containing 5 μl Qiagen Multiplex Buffer, 1 μl primer-mix, 3 μl Milli-Q water and 1 μl diluted DNA extract.

PCR products were diluted 1:99 with Milli-Q water and length separated on an ABI Prism® 3130 XL Genetic analyzer (Applied Biosystems) using fluorescently labeled primers. Allele sizes were determined using ABI Prism® GeneMapper™ Software version 4.0 (Applied Biosystems).

Paternity analyses were run with Cervus version 3.0.7 [54]. For the simulation of parentage, we used 4 candidate fathers, of which 75% were sampled, and 10000 offspring, with an error rate in likelihood calculations of 0.01. For paternity assignments, we set the limit to two or fewer mismatches for a male to be considered the true sire of his offspring, and a proportion of alleles shared of at least 0.875. The combined exclusion probability for the markers was > 99.99% for both the 8-microsatellite panel and the 22-microsatellite panel.

For all three years combined, a total of 20 samples did not amplify in PCR, and were excluded from the analyses. For 16 chicks in three broods, the social father was not identified, so these broods were excluded from analyses of within pair and extra-pair success. Chicks from these nests who were successfully assigned to a father were included in analyses of total reproductive success. Two males were most likely polygynous, which could alter the likelihood of being cuckolded. Their nests were thus excluded from analyses of within-pair success (20 chicks). In one additional nest (6 chicks), the genetic sire had not had a sperm sample taken in that year, and this nest was excluded from analyses. For three nests, the social mother was not sampled, but paternity analysis was still conducted without a known mother.

### Statistical analyses

Some of the males were measured multiple times within the same season, but we only used one measurement per male per year, except in repeatability analyses. We used the measurement with the highest number of good motile tracks, to obtain the most accurate averages possible. In analyses where we test for associations between different variables and paternity success, we account for the presence of the same males in two years by keeping both recordings and including male identity (ring number) as a random variable in our models. We checked our results by running each test without the second recordings, but this did not change any of our conclusions qualitatively. For correlation analyses between different male characteristics, we only kept one measurement per male (the first year). We centered all of the variables to the mean of each year separately, as many of them were significantly different between years. In analyses reported in the main text, we tested individual sperm measures against paternity success, but we also ran a principal component analysis (PCA) to combine all sperm characteristics into fewer variables, and ran the two components with the highest eigenvalues against all measures of paternity success. We used three different measures of paternity success in our analyses: 1) within-pair fertilization success (i.e. sired all offspring in his own social nest or was cuckolded at least once), 2) extra-pair fertilization success (i.e. sired at least one offspring in another male’s nest or did not), and 3) total number of offspring sired (the number of sired offspring in the social nest plus the number of offspring sired in other nests). For the first two measures, we used generalized linear mixed models with binomial error distributions and bobyqa optimization [55], and for the third we used linear mixed models with normal distributions, to test for possible associations between different variables and paternity success. We included red border width and age as covariates in all models relating sperm characteristic to paternity success, since controlling for correlated variables is necessary to properly test how traits correlate with fitness [47]. For total sperm length, we included the quadratic term, in addition to the linear, in our models to test for the possibility of stabilizing selection on sperm length [32]. To further explore results, we also tested whether sperm characteristics predicted the number of extra pair offspring sired and the proportion of chicks sired in extra pair nests, among only those males that sired extra-pair chicks, using Spearman's correlation tests. It should be noted that measurements of extra-pair success are more prone to measurement error, compared to measurements of within-pair success, since we do not have complete control of all nests in the area. We present the results of these analyses in the supplementary (S2 Table).

Regressing traits—here, sperm measurements—on fitness is considered to be selection analysis [47], and such tests are therefore central to understanding whether traits are evolving via selection. When no relationship between traits and fitness are observed, the absence of selection can reflect insufficient opportunity for selection, due to too little variation in fitness measures among individuals. We assessed the opportunity for selection in our population by calculating standardized variance in total male reproductive success, a widely accepted measure. That is, for each year separately, we divided standardized variance by its squared mean [56, 57].

In addition to testing sperm characteristics in separate models, we performed a PCA to combine all sperm characteristics into a few synthetic components. The first two components explained 99.999999% of the variation and were tested against all three measures of paternity success. The results were qualitatively similar to the analyses on single components and can be found as supplementary material (S3 Table).

We also directly compared pairs consisting of within-pair males and the extra-pair males who had cuckolded them in paired t-tests (*N* = 15 pairs, 26 individual males). Some of the males were cuckolded or cuckolders several times, but as all of the male dyads were different, we kept all as independent data points.

To assess how reliably we could use a single sperm sample as indicative of sperm characteristics at the time of fertilization, we investigated the repeatability of the variables. We tested repeatability separately among and within years by comparing the males who had been sampled in two or three years (*N* = 10), and the males who had been measured multiple times in the same year (*N* = 19 for the proportion of motile sperm, *N* = 13 for sperm velocity, *N* = 14 for sperm morphometry). We ran linear regression models between first and second measurement of all variables to find the correlation values [58]. Within-season, all sperm component lengths and derived sperm morphology variables were highly repeatable, whereas sperm motility measures had low repeatability (Table 1). Between years, sperm component lengths were quite highly repeatable, whereas sperm motility measures again had very low repeatability (Table 2).

**Table 1:** Within-season repeatability, comparing measurements of males that have been sampled twice in the same year^a^. *R*^2^ is the repeatability, mean ± SE is shown for first and second measure, along with *F* value, number of males (*N*), and *p* value. All significant correlations (*p* < 0.05) were robust to correction for multiple testing using false discovery rate correction [59], and are marked in bold.

Variables	*R*^2^	Mean ± SE		*F*	*N*	*p*
		1st measure	2nd measure			
**Sperm morphology**						
Head length (μm)	0.65	15.51 ± 0.14	15.55 ± 0.13	22.23	14	**0.0005**
Midpiece length (μm)	0.93	175.81 ± 2.02	175.67 ± 1.95	148.48	14	**4.08E-08**
Tail length (μm)	0.93	16.87 ± 1.47	16.8 ± 1.35	160.94	14	**2.60E-08**
Flagellum length (μm)	0.93	192.68 ± 1.45	192.46 ± 1.47	162.19	14	**2.49E-08**
Total sperm length (μm)	0.93	208.18 ± 1.55	208.01 ± 1.54	170.76	14	**1.86E-08**
F:H^b^	0.51	12.46 ± 0.08	12.41 ± 0.10	12.32	14	**0.004**
M:TSL^c^	0.93	0.84 ± 0.01	0.84 ± 0.01	149.16	14	**3.97E-08**
**Sperm motility**						
Velocity (μm/s)	0.10	140.94 ± 9.85	131.15 ± 10.17	2.23	13	0.15
Proportion motile	1.86E-06	0.23 ± 0.05	0.21 ± 0.04	4.47E-05	19	0.99

^a^ Many of the repeated measurements were taken on the same day (sperm morphology: 9/14, sperm velocity: 12/13, proportion motile: 15/19).

^b^ Flagellum to head ratio.

^c^ Midpiece to total sperm length.

**Table 2:** Between-year repeatability, comparing measurements of males sampled in both years. *R*^2^ is the repeatability, mean ± SE is shown for the first year of capture (2013 or 2014) and the second year of capture (2014 or 2015), along with *F* value, number of males (*N*) and *p* value. All significant correlations (*p* < 0.05) were robust to correction for multiple testing using false discovery rate correction [59], and are marked in bold.

Variables	*R*^2^	Mean ± SE		*F*	*N*	*p*
		1st year	2nd year			
**Sperm morphology**						
Head length (μm)	0.78	15.68 ± 0.16	15.74 ± 0.20	28.50	10	**0.0007**
Midpiece length (μm)	0.65	175.81 ± 2.75	175.02 ± 4.55	15.13	10	**0.005**
Tail length (μm)	0.80	16.16 ± 1.63	15.70 ± 2.03	32.62	10	**0.0004**
Flagellum length (μm)	0.52	191.97 ± 1.92	190.72 ± 2.93	8.78	10	**0.02**
Total sperm length (μm)	0.53	207.65 ± 1.93	206.46 ± 3.01	8.94	10	**0.02**
F:H^a^	0.68	12.28 ± 0.18	12.15 ± 0.20	16.78	10	**0.003**
M:TSL^b^	0.75	0.85 ± 0.01	0.85 ± 0.01	23.57	10	**0.001**
**Sperm motility**						
Velocity (μm/s)	0.05	144.86 ± 12.75	150.73 ± 11.48	0.35	9	0.57
Proportion motile	0.02	0.37 ± 0.10	0.44 ± 0.08	0.16	10	0.70

^a^ Flagellum to head ratio.

^b^ Midpiece to total sperm length

We used R version 3.2.2 for all statistical analyses (R core team, 2016). Residuals from t-tests and linear mixed models that assumed normality were checked by eye.

## Results

### Patterns of parentage

In 2013, 21% (6/29) of broods contained extra-pair offspring and 10% (16/167) of offspring were sired by extra-pair males. In 2014, 19% (3/16) of broods contained extra-pair offspring and 5% (4/76) of offspring were sired by extra-pair males. In 2015, 38% (6/16) of broods contained extra-pair offspring and 18% (13/72) of offspring were sired by extra-pair males.

Seven of the 15 broods where cuckoldry occurred had more than one extra-pair offspring, and two males experienced total loss of within-pair paternity. Both social fathers were observed feeding the chicks, thus confirming that they were in fact the social males. We identified 16 males who had sired a total of 70% (23/33) of the extra-pair young. Seven of these had nests in our study area, and none of them had been cuckolded in their own nest. Nevertheless, cuckolders were not significantly less likely to be cuckolded than non-cuckolders for all years combined (two-tailed Fisher’s exact test: *p* = 0.18).

In total, including nests with unknown social fathers, we assigned the sires of 93% (331/357) of the offspring. Hence, we lack information on 7% (26/357) of the offspring, and males may also have sired additional offspring outside of the study area. However, these missing data are unlikely to bias our results, as they may be random with respect to sperm characteristics.

### Sperm characteristics and paternity success

Mean ± SD sperm length was 210.32 ± 5.76 μm; sperm velocity was 149.47 ± 31.21 μm/sec.

**Fig 1:**
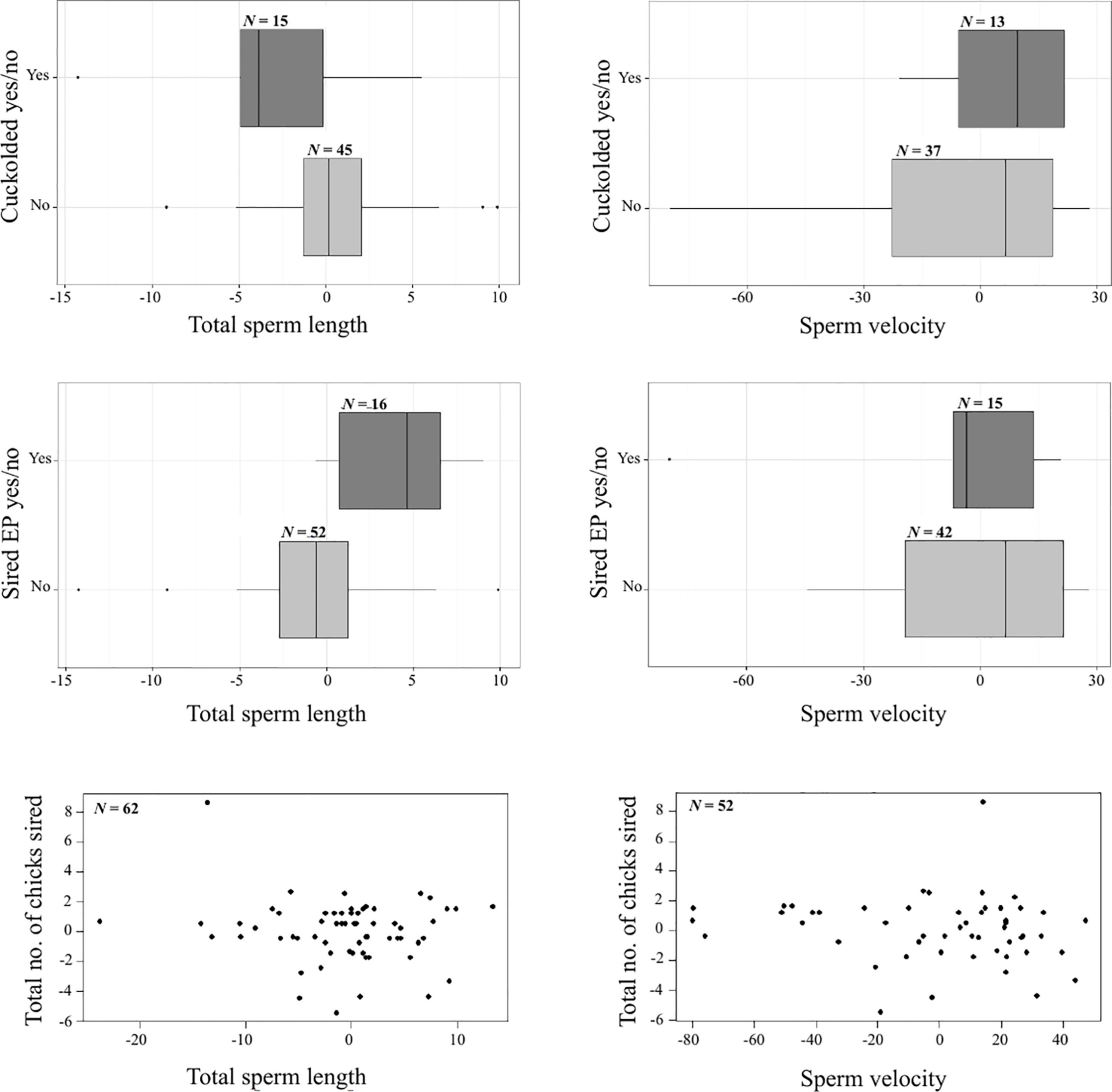
Sperm characteristics and paternity success. Total sperm length (left) and sperm velocity (right) compared to three measures of fertilization success: within-pair (WP) fertilization success (top two plots), extra-pair (EP) fertilization success (middle two plots), and total fertilization success (total number of offspring sired; bottom two plots). The variables have been centered to the mean of each year. In the boxplots, the left and the right of the boxes are the first and third quartiles, and the line inside the box is the median. The whiskers represent the lowest and highest points still within the 1.5 interquartile range, and dots outside of the whiskers are outliers.

Neither sperm morphology nor sperm motility correlated significantly with within-pair fertilization success, extra-pair fertilization success or total number of offspring sired (Table 3, Fig 1). Red border width and age were added as covariates to the model, but none of these correlations were significant (S4 Table). The results were qualitatively the same in models that did not control for red border and age (data not shown), and, similarly, models using principal components combining sperm traits (S3 Table) and Spearman’s correlations between different measures of extra-pair success and sperm traits (S2 Table) were non-significant. There was neither a linear nor a quadratic relationship between total sperm length and paternity success (Table 3). Thus, there appeared to be no evidence of selection acting on sperm traits in the population. The opportunity for selection was calculated as 0.11, 0.10, and 0.29 in 2013, 2014, and 2015, respectively.

**Table 3:** Correlations between sperm characteristics and fertilization success in generalized linear mixed models. Fertilization success was measured as within-pair (WP) fertilization success (males that had not been cuckolded = 0; males that had been cuckolded = 1), extra-pair (EP) fertilization success (males that had not sired extra-pair offspring = 0; males that had sired extra-pair offspring = 1), and total fertilization success (total number of offspring sired). Red border width and age were added as covariates to the models, but their results are not shown here (see S4 Table).

	WP fertilization success		EP fertilization success		Total fertilization success	
	Estimate ± SE	*Z (p)*	Estimate ± SE	*Z (p)*	Estimate ± SE	*t (p)*
**Sperm morphology**^**a**^						
Head length (μm)	0.23 ± 0.71	0.33 (0.74)	0.88 ± 0.83	1.06 (0.29)	0.24 ± 0.64	0.37 (0.71)
Midpiece length (μm)	-0.03 ± 0.04	-0.79 (0.43)	0.01 ± 0.04	0.12 (0.91)	-0.04 ± 0.03	-1.20 (0.24)
Tail length (μm)	-0.001 ± 0.07	-0.02 (0.99)	0.08 ± 0.07	1.25 (0.21)	0.06 ± 0.06	0.97 (0.34)
Flagellum length (μm)	-0.06 ± 0.05	-1.03 (0.30)	0.09 ± 0.06	1.56 (0.12)	-0.04 ± 0.04	-0.87 (0.39)
Total sperm length (μm): linear	-0.06 ± 0.06	-0.99 (0.32)	0.08 ± 0.06	1.36 (0.17)	-0.01 ± 0.05	-0.29 (0.77)
Total sperm length (μm): quadratic	-0.002 ± 0.01	-0.24 (0.81)	0.01 ± 0.01	1.33 (0.18)	0.003 ± 0.004	0.85 (0.40)
F:H^b^	-0.78 ± 0.71	-1.09 (0.27)	0.29 ± 0.68	0.43 (0.67)	-0.59 ± 0.58	-1.01 (0.32)
M:TSL^c^	-2.59 ± 13.33	-0.19 (0.85)	-16.03 ± 14.21	-1.13 (0.26)	-13.28 ± 11.76	-1.13 (0.26)
**Sperm motility**						
Velocity^d^ (μm/s)	0.02 ± 0.01	1.27 (0.20)	0.001 ± 0.01	0.04 (0.97)	-0.01 ± 0.01	-0.92 (0.45)
Proportion motile^e^	-0.91 ± 1.74	-0.52 (0.60)	-1.73 ± 1.90	-0.91 (0.36)	-2.02 ± 1.59	-1.28 (0.21)

^a^ Sperm morphology: *N* = 60/68/62.

^b^ Flagellum to head ratio.

^c^ Midpiece to total sperm length.

^d^ Velocity: *N* = 50/57/52.

^e^ Proportion motile: *N* = 56/64/58.

**Fig 2:**
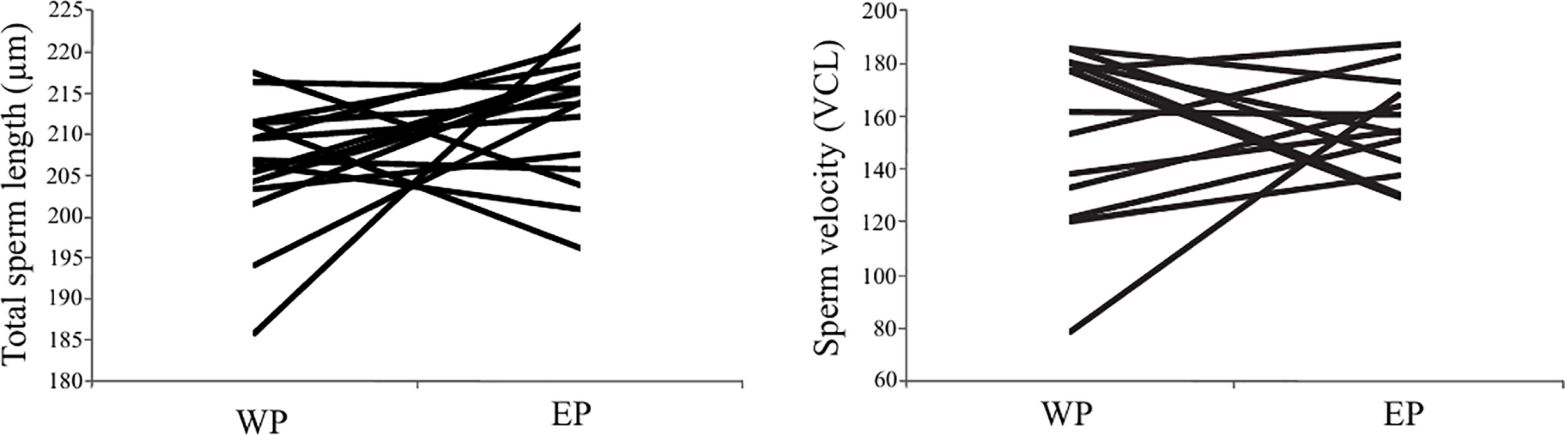
Paired comparisons of total sperm length (left) and sperm velocity (VCL; right) between within-pair (WP) males and the extra-pair (EP) males that cuckolded them.

There were no significant differences between within-pair males and the males that cuckolded them in paired comparisons (Table 4, Fig 2).

**Table 4:** Paired comparisons of within-pair (WP) males and the extra-pair (EP) male that cuckolded them, with mean values (± SE) of sperm traits and body morphology traits. Uncorrected *p* values are shown.

Variables	WP	EP	*t*	*N* pairs	*p*
**Sperm morphology**					
Head length (μm)	15.78 ± 0.15	15.87 ± 0.16	-0.55	15	0.59
Midpiece length (μm)	173.21 ± 3.06	177.91 ± 2.27	-1.29	15	0.22
Tail length (μm)	17.30 ± 1.59	18.13 ± 2.16	-0.42	15	0.68
Flagellum length (μm)	190.51 ± 2.00	196.04 ± 1.89	-1.66	15	0.12
Total sperm length (μm)	206.30 ± 2.10	211.91 ± 1.96	-1.66	15	0.12
F:H^a^	12.10 ± 0.10	12.39 ± 0.14	-1.39	15	0.19
M:TSL^b^	0.84 ± 0.01	0.84 ± 0.01	-0.11	15	0.91
**Sperm motility**					
Velocity (μm/s)	153.44 ± 9.17	156.67 ± 5.12	-0.30	13	0.77
Proportion motile	0.21 ± 0.05	0.25 ± 0.06	-0.65	14	0.53

^a^ Flagellum to head ratio.

^b^ Midpiece to total sperm length.

### Sperm characteristics and other male traits

There were no significant differences between the two age groups in total sperm length (*N* = 102, *W* = 1387, *p* = 0.22) or sperm swimming speed (*N* = 91, *W* = 855, *p* = 0.31). There was no significant correlation between red border width and total sperm length or sperm swimming speed (Table 5), nor were there any significant correlations between these sperm characteristics and tarsus length, wing length or mass (Table 5).

**Table 5:** Estimated slope relating male morphological characters to sperm characteristics in generalized linear mixed models. The table shows uncorrected p-values; none were significant after correcting for multiple testing using false discovery rate correction [59].

	Total sperm length (μm)			Sperm velocity (μm/s)		
	Estimate ± SE	*t (p)*	*N*	Estimate ± SE	*t (p)*	*N*
Red border width	-0.12 ± 0.30	-0.41 (0.69)	100	1.57 ± 1.60	0.98 (0.33)	91
Tarsus length	1.39 ± 0.71	1.96 (0.05)	100	-3.36 ± 3.83	-0.88 (0.38)	91
Wing length	0.57 ± 0.32	1.76 (0.08)	100	1.17 ± 1.73	0.68 (0.50)	91
Mass	1.57 ± 0.77	2.02 (0.05)	98	0.43 ± 4.13	0.11 (0.92)	90

## Discussion

We found no significant correlations between characteristics of the males’ sperm and paternity success, external morphology or age.

Our first aim was to test the hypothesis that sperm characters are related to fertilization success. We found no evidence to support this hypothesis, despite having high sample sizes for sperm morphology comparisons, and moderate sample sizes for sperm velocity measures. So far, most studies that have investigated correlations between sperm traits and paternity success within a passerine species have not found evidence of directional selection on sperm length ([23, 35, 36], this study). However, in an experimental study on captive zebra finches, Bennison et al. [22] found that males with longer sperm had higher fertilization success, and some comparative studies in passerines indicate that species with higher rates of extra-pair paternity have longer sperm [12,13]. Similarly, interspecific studies have shown that the between-male variation in sperm length is negatively correlated with the frequency of extra-pair paternity [13,31,32]. Since sperm competition can act as a stabilizing selection pressure, it is possible that there is a non-linear (quadratic) relationship between sperm length and paternity success. We did not find such a relationship (see also Cramer et al. [36]).

Sperm velocity was not correlated with paternity success, corroborating the findings of Laskemoen et al. [23] in tree swallows, the only other study on sperm velocity in a wild passerine population. The repeatability of sperm swimming speed across repeated measures of the same male was low in our population, suggesting that a single assessment of sperm swimming velocity, under field conditions, may not provide sufficient information for testing how sperm swimming speed affects fertilization success. Additionally, sperm swimming speed may be a less important competitive trait in animals with sperm storage by females; for example, in birds, the sperm cells may just be passively transported by the female from the SSTs to the site of fertilization [60]. How sperm storage by the female affects sperm swimming speed is poorly understood [61,62]. However, in controlled laboratory experiments, swimming speed has been found to affect fertilization success in birds [15,18].

Our results, and the results of previous work in wild birds [23,35,36], do not corroborate the results of comparative and lab studies [12,13,22]. This may be in part because in natural settings, other factors such as the order in which males copulate with the female or the relative number of sperm cells each of them transfer may be more important. Laskemoen et al. [23] found sperm quantity to be the most important predictor of fertilization success in a study on tree swallows. Other studies have found that copulation order is an important predictor of fertilization success [24,63]. However, extra-pair males do not appear to time inseminations better than within-pair males in bluethroats [64]. Unfortunately, we do not have measures on sperm quantity or copulation order in our study. Cryptic female preference for sperm with compatible genotypes may also make it difficult to detect selection on sperm morphology and velocity. Extra-pair bluethroat offspring have been found to be more heterozygous and have higher immunocompetence than their within-pair half siblings, likely because extra-pair mates are on average less genetically similar to the female than within-pair mates [41,42]. Thus, cryptic female choice may play a significant role in postcopulatory sexual selection in this species.

Between-male variation in sperm morphology was moderate to low in our population, which theoretically could reduce the statistical power to detect associations between these variables and paternity success. However, reduced variation in sperm parameters correlates with higher opportunity for postcopulatory sexual selection across species [30–32], suggesting that species with the lowest between-male variation in sperm traits may also be the species where the strongest—and easiest-to-detect—selection can occur. We note that, of the four previous studies on wild passerines, selection on sperm traits was detected in the two species with the highest rates of extra-pair paternity and low to moderate between-male variation in sperm morphology, but was not found in the two species with relatively lower extra-pair paternity rates and higher variation in sperm morphology (Table 6). The relatively low EPP rates in our bluethroat population in the years of our study are more similar to the latter two species. As such, it is perhaps not surprising that we did not find a relationship between sperm characteristics and paternity success in this study.

**Table 6:** Summary of species where the relationship between sperm morphology and paternity success has been assessed in the wild. For each species, we report the between-male variation in sperm total length (expressed as the coefficient of variation, CV_bm_), the percent of offspring sired by an extra-pair male (EPY), the percent of broods containing at least one extra-pair offspring (EPB), and whether published works found significant relationships between sperm morphology and paternity. Where more than one published source is available for estimates of EPY and EPB, we preferentially use the estimate from the population and/or year where CV_bm_ was assessed. Our sources are as follows: Tree swallow: [23, 65], Superb fairywren: [35, 66], Collared flycatcher: [37, 43], Bluethroat: this study, House wren: [36, 67, 68].

Species	CV_bm_	% EPY, % EPB	Significantly related?
Tree swallow	1.75	51, 86^1^	yes
Superb fairywren	2.10	67–76, 65–95	yes
Collared flycatcher	2.40	19, NA^2^	no
Bluethroat	2.73	10.5, 24.6	no
House wren	4.60^3^	14, 38	no

^1^Here we report the values for the subset of individuals analyzed for CV_bm_.

^2^Missing information for EPB.

^3^Average of three annual values.

Our second aim was to investigate whether sperm characters are related to male traits that covary with fertilization success [69]. The width of the red border did not correlate with sperm characteristics in this study. Since red border width previously has been found to correlate with within-pair fertilization success [39] it may be a factor in precopulatory sexual selection which may not be reflected in higher sperm competitive abilities. Male age has been found to correlate with extra-pair fertilization success in previous studies (e.g. [70–72]), including in bluethroats [39]. In the study by Johnsen et al. [39], old males did not have higher within-pair fertilization success than young males, suggesting that old males are not generally preferred. Old males might be better at courting females and they spend less time guarding their mates than young males, meaning that they are more available for extra-pair copulations [72]. It is also possible that older males do better in sperm competition as they may be able to produce more sperm [40]. However, this may not be reflected in higher sperm quality. Other male characteristics that might be under precopulatory selection, and which might obscure postcopulatory selection on sperm traits, were not measured.

The levels of extra-pair paternity found in this study are lower than the average levels found in 12 years of research on this population. On average in previous studies, 44% of broods contained extra-pair offspring, and 23% of offspring were extra-pair [38,42]. In contrast, 25% of broods and 10% of offspring were extra-pair in this data set. Such annual fluctuations in levels of extra-pair paternity are not well understood, but may be related to weather conditions during the fertile period [38]. Likely due to this reduced level of EPP, the opportunity for selection was relatively low in two years of this study, in comparison with either other years in the same study population (e.g., opportunity for selection was 0.38 in 1998 and 0.31 in 1999, two years with higher EPP rates) or in comparison to some other species (e.g., 0.49 in polygynous red-winged blackbirds, *Agelaius phoeniceus* [73]; 0.68–0.74 in black-throated blue warblers, *Setophaga caerulescens* [74]; see also review in Table 1, Freeman-Gallant et al. 2005 [75]). This stochastic low opportunity for selection in two of the years of our study may have limited our ability to detect significant relationships between sperm characteristics and total reproductive success. Our sample sizes (in terms of number of males) are similar to Laskemoen et al. [23] and Calhim et al. [35], who found significant correlations between sperm traits and paternity success, but the relatively small number of nests with extra-pair paternity may have affected our ability to detect subtle effects of sperm traits on paternity.

## Conclusion

We found no evidence for associations between sperm morphology or velocity and paternity success in bluethroats, indicating that there are other factors that affect male fertilization success more strongly. Such factors might include sperm quantity, cryptic female choice of males with compatible genes, or selection for precopulatory traits that are not correlated with sperm morphology or swimming speed. Despite strong evidence for selection on sperm morphology and velocity from interspecific comparative studies and from experimental work, detecting selection on sperm morphology and velocity in wild populations remains a challenge.

## Supporting information

S1 Table PCR marker characteristics. Characteristics of the 22 markers that were used in PCR, where *k* is the number of alleles, *N* is the number of adult individuals, HObs is the observed heterozygosity and F(Null) is the estimated frequency of null alleles. Finally we have listed the allelic richness of each marker, and their concentration (μl of 100 μMolar stock per PCR). The eight markers that were singled out in 2015 are in bold.
(DOCX)

S2 Table Spearman's correlation analyses. Further analyses relating sperm characteristics to success in siring extra-pair offspring, among males that sired extra-pair offspring only. One male had sperm data and sired offspring in two years; we included only the first year’s data to avoid pseudoreplication. Results were qualitatively the same if we excluded the second year or treated the two years as independent. Note that measurements of extra-pair success are more prone to measurement error, compared to measurements of within-pair success, since we do not have complete control of all nests in the area.
(DOCX)

S3 Table Principal component analysis. Correlations between the two components with the highest eigenvalues from a Principal component analysis (PCA) of all sperm measurements, and three measures of fertilization success in generalized linear mixed models, with red border width and age included as covariates. Fertilization success was measured as within-pair (WP) fertilization success (males that had not been cuckolded = 0; males that had been cuckolded = 1), extra-pair (EP) fertilization success (males that had not sired extra-pair offspring = 0; males that had sired extra-pair offspring = 1), and total fertilization success (total number of offspring sired).
(DOCX)

S4 Table Expanded Table 3. Correlations between sperm characteristics and fertilization success in generalized linear mixed models, with red border width and age included as covariates. Fertilization success was measured as within-pair (WP) fertilization success (males that had not been cuckolded = 0; males that had been cuckolded = 1), extra-pair (EP) fertilization success (males that had not sired extra-pair offspring = 0; males that had sired extra-pair offspring = 1), and total fertilization success (total number of offspring sired).
(DOCX)
