# Supplementary material for: Sperm morphology, sperm motility and paternity success in the bluethroat (Luscinia svecica)
Source: PLoS One. 2018 Mar 6;13(3):e0192644. doi: 10.1371/journal.pone.0192644 (PMC5839561; doi:10.1371/journal.pone.0192644)
Supplement: S1 Table — Characteristics of the 22 markers that were used in PCR, where k is the number of alleles, N is the number of adult individuals, HObs is the observed heterozygosity and F(Null) is the estimated frequency of null alleles. Finally we have listed the allelic richness of each marker, and their concentration (μl of 100 μMolar stock per PCR). The eight markers that were singled out in 2015 are in bold. (DOCX) [file pone.0192644.s001.docx]

**S1 Table. PCR marker characteristics.** Characteristics of the 22 markers that were used in PCR, where *k* is the number of alleles, *N* is the number of adult individuals, HObs is the observed heterozygosity and F(Null) is the estimated frequency of null alleles. Finally we have listed the allelic richness of each marker, and their concentration (µl of 100 µMolar stock per PCR). The eight markers that were singled out in 2015 are in bold.

| **Panel** | **Locus** | ***k*** | ***N*** | **HObs** | **F(Null)** | **Allelic r.** | **Concentration** |
| --- | --- | --- | --- | --- | --- | --- | --- |
| 1 | ZF-C59 | 7 | 79 | 0.506 | 0.0130 | 6.92 | 0.08 |
| **1** | **EST9^1^** | **35** | **78** | **0.949** | **-0.0026** | **29.48** | **0.015** |
| 1 | GG-C25 | 7 | 79 | 0.709 | 0.0353 | 6.92 | 0.015 |
| 1 | ZF-S8 | 6 | 79 | 0.443 | 0.0407 | 5.92 | 0.015 |
| **1** | **ZF-S9^1^** | **13** | **79** | **0.823** | **0.0145** | **12.77** | **0.06/0.03** |
| 2 | EST46 | 11 | 80 | 0.800 | -0.0158 | 10.82 | 0.02 |
| **2** | **FH310^1^** | **26** | **80** | **0.838** | **0.0065** | **24.09** | **0.02** |
| 2 | FH350 | 5 | 79 | 0.304 | -0.0102 | 5.00 | 0.03 |
| 2 | FH361 | 6 | 80 | 0.438 | -0.0131 | 5.91 | 0.03 |
| **2** | **FH407^1^** | **23** | **80** | **0.925** | **-0.0109** | **22.53** | **0.03** |
| 3 | FH413 | 3 | 80 | 0.425 | 0.0777 | 3.00 | 0.03 |
| **3** | **FH408^2^** | **33** | **80** | **0.913** | **0.0155** | **32.01** | **0.04** |
| 3 | FH403 | 16 | 80 | 0.700 | 0.0693 | 15.55 | 0.03 |
| 3 | FH356 | 3 | 80 | 0.263 | 0.0923 | 3.00 | 0.03 |
| 4 | FH431 | 12 | 80 | 0.713 | 0.0332 | 11.72 | 0.02 |
| **4** | **FH448^2^** | **20** | **80** | **0.825** | **0.0465** | **19.71** | **0.02** |
| 4 | FH452 | 8 | 80 | 0.538 | 0.0184 | 7.82 | 0.02 |
| **4** | **FH465^2^** | **27** | **80** | **0.900** | **0.0047** | **26.44** | **0.10** |
| **4** | **FH466^2^** | **11** | **80** | **0.763** | **0.0074** | **10.72** | **0.04/0.06** |
| 5 | FH227 | 9 | 75 | 0.573 | 0.0484 | 8.95 | 0.02 |
| 5 | FH230 | 13 | 75 | 0.840 | -0.0056 | 12.87 | 0.04 |
| 5 | FH359 | 16 | 75 | 0.880 | 0.0185 | 15.97 | **0.03** |

^1^ These markers were combined in panel 1 in 2015
^2^ These markers were combined in panel 2 in 2015
