# Supplementary material for: Sperm morphology, sperm motility and paternity success in the bluethroat (Luscinia svecica)
Source: PLoS One. 2018 Mar 6;13(3):e0192644. doi: 10.1371/journal.pone.0192644 (PMC5839561; doi:10.1371/journal.pone.0192644)
Supplement: S2 Table — Further analyses relating sperm characteristics to success in siring extra-pair offspring, among males that sired extra-pair offspring only. One male had sperm data and sired offspring in two years; we included only the first year’s data to avoid pseudoreplication. Results were qualitatively the same if we excluded the second year or treated the two years as independent. Note that measurements of extra-pair success are more prone to measurement error, compared to measurements of within-pair success, since we do not have complete control of all nests in the area. (DOCX) [file pone.0192644.s002.docx]

**S2 Table. Spearman's correlation analyses.** Further analyses relating sperm characteristics to success in siring extra-pair offspring, among males that sired extra-pair offspring only. One male had sperm data and sired offspring in two years; we included only the first year’s data to avoid pseudoreplication. Results were qualitatively the same if we excluded the second year or treated the two years as independent. Note that measurements of extra-pair success are more prone to measurement error, compared to measurements of within-pair success, since we do not have complete control of all nests in the area.

|  | | |
| --- | --- | --- |
|  | Estimated Spearman's rho (*p*) | |
| Predictor variable | Number of EPY sired | Proportion of EPY sired in EP nest |
| Head length (μm) (*N* = 14) | 0.13 (0.67) | -0.06 (0.85) |
| Midpiece length (μm) (*N* = 14) | 0.05 (0.86) | -0.34 (0.24) |
| Tail length (μm) (*N* = 14) | -0.16 (0.59) | 0.24 (0.40) |
| Flagellum length (μm) (*N* = 14) | -0.20 (0.50) | -0.19 (0.51) |
| Total sperm length (μm) (*N* = 14) | -0.20 (0.50) | -0.19 (0.51) |
| F:H^a^ (*N* = 14) | -0.13 (0.67) | -0.16 (0.58) |
| M:TSL^b^ (*N* = 14) | 0.20 (0.50) | -0.24 (0.40) |
| Velocity (µm/s) (*N* = 13) | 0.23 (0.44) | 0.44 (0.13) |
| Proportion motile (*N* = 14) | 0.17 (0.57) | 0.45 (0.11) |
| CVwm^c^ (*N* = 14) | 0.28 (0.34) | 0.13 (0.65) |

^a^ Flagellum to head ratio
^b^ Midpiece to total sperm length
^c^ Within-male coefficient of variation in total sperm length. CV = (standard deviation/mean) * 100
