# Supplementary material for: Sperm morphology, sperm motility and paternity success in the bluethroat (Luscinia svecica)
Source: PLoS One. 2018 Mar 6;13(3):e0192644. doi: 10.1371/journal.pone.0192644 (PMC5839561; doi:10.1371/journal.pone.0192644)
Supplement: S3 Table — Correlations between the two components with the highest eigenvalues from a Principal component analysis (PCA) of all sperm measurements, and three measures of fertilization success in generalized linear mixed models, with red border width and age included as covariates. Fertilization success was measured as within-pair (WP) fertilization success (males that had not been cuckolded = 0; males that had been cuckolded = 1), extra-pair (EP) fertilization success (males that had not sired extra-pair offspring = 0; males that had sired extra-pair offspring = 1), and total fertilization success (total number of offspring sired). (DOCX) [file pone.0192644.s003.docx]

**S3 Table. Principal component analysis.** Correlations between the two components with the highest eigenvalues from a Principal component analysis (PCA) of all sperm measurements, and three measures of fertilization success in generalized linear mixed models, with red border width and age included as covariates. Fertilization success was measured as within-pair (WP) fertilization success (males that had not been cuckolded = 0; males that had been cuckolded = 1), extra-pair (EP) fertilization success (males that had not sired extra-pair offspring = 0; males that had sired extra-pair offspring = 1), and total fertilization success (total number of offspring sired).

|  | **WP fertilization success** | | **EP fertilization success** | | **Total fertilization success** | |
| --- | --- | --- | --- | --- | --- | --- |
|  | **Estimate  ± SE** | ***Z (p)*** | **Estimate  ± SE** | ***Z (p)*** | **Estimate**  **± SE** | ***t (p)*** |
| PC1 | 0.02 ± 0.01 | 1.03 (0.30) | 0.003 ± 0.01 | 0.19 (0.85) | -0.01 ± 0.01 | 2.13 (0.44) |
| PC2 | 0.02 ± 0.03 | 0.59 (0.56) | -0.01 ± 0.03 | -0.32 (0.75) | 0.03 ± 0.01 | 3.65 (0.13) |
