# Supplementary material for: Sperm morphology, sperm motility and paternity success in the bluethroat (Luscinia svecica)
Source: PLoS One. 2018 Mar 6;13(3):e0192644. doi: 10.1371/journal.pone.0192644 (PMC5839561; doi:10.1371/journal.pone.0192644)
Supplement: S4 Table — Correlations between sperm characteristics and fertilization success in generalized linear mixed models, with red border width and age included as covariates. Fertilization success was measured as within-pair (WP) fertilization success (males that had not been cuckolded = 0; males that had been cuckolded = 1), extra-pair (EP) fertilization success (males that had not sired extra-pair offspring = 0; males that had sired extra-pair offspring = 1), and total fertilization success (total number of offspring sired). (DOCX) [file pone.0192644.s004.docx]

**S4 Table. Expanded Table 3.** Correlations between sperm characteristics and fertilization success in generalized linear mixed models, with red border width and age included as covariates. Fertilization success was measured as within-pair (WP) fertilization success (males that had not been cuckolded = 0; males that had been cuckolded = 1), extra-pair (EP) fertilization success (males that had not sired extra-pair offspring = 0; males that had sired extra-pair offspring = 1), and total fertilization success (total number of offspring sired).

|  | **WP fertilization success** | | **EP fertilization success** | | | **Total fertilization success** | |
| --- | --- | --- | --- | --- | --- | --- | --- |
|  | **Estimate  ± SE** | ***Z (p)*** | | **Estimate  ± SE** | ***Z (p)*** | **Estimate**  **± SE** | ***t (p)*** |
| **Sperm morphology^a^** |  |  | |  |  |  |  |
| Head length (µm) | 0.23 ± 0.71 | 0.33 (0.74) | | 0.88 ± 0.83 | 1.06 (0.29) | 0.24 ± 0.64 | 0.37 (0.71) |
| + age | 0.09 ± 0.64 | 0.14 (0.89) | | 0.32 ± 0.72 | 0.45 (0.65) | 0.49 ± 0.58 | 0.83 (0.41) |
| + red border width | 0.06 ± 0.14 | 0.45 (0.65) | | -0.17 ± 0.16 | -1.05 (0.29) | -0.03 ± 0.13 | -0.19 (0.85) |
| Midpiece length (µm) | -0.03 ± 0.04 | -0.79 (0.43) | | 0.01 ± 0.04 | 0.12 (0.91) | -0.04 ± 0.03 | -1.20 (0.24) |
| + age | 0.06 ± 0.64 | 0.10 (0.92) | | 0.24 ± 0.69 | 0.35 (0.73) | 0.40 ± 0.58 | 0.69 (0.49) |
| + red border width | 0.06 ± 0.14 | 0.44 (0.66) | | -0.19 ± 0.16 | -1.17 (0.24) | -0.02 ± 0.13 | -0.17 (0.87) |
| Tail length (µm) | -0.001 ± 0.07 | -0.02 (0.99) | | 0.08 ± 0.07 | 1.25 (0.21) | 0.06 ± 0.06 | 0.97 (0.34) |
| + age | 0.07 ± 0.63 | 0.12 (0.91) | | 0.30 ± 0.74 | 0.41 (0.68) | 0.45 ± 0.58 | 0.78 (0.44) |
| + red border width | 0.06 ± 0.14 | 0.43 (0.66) | | -0.20 ± 0.17 | -1.24 (0.22) | -0.02 ± 0.13 | -0.17 (0.87) |
| Flagellum length (µm) | -0.06 ± 0.05 | -1.03 (0.30) | | 0.09 ± 0.06 | 1.56 (0.12) | -0.04 ± 0.04 | -0.87 (0.39) |
| + age | 0.06 ± 0.64 | 0.10 (0.92) | | 0.17 ± 0.66 | 0.26 (0.80) | 0.41 ± 0.58 | 0.71 (0.48) |
| + red border width | 0.07 ± 0.14 | 0.48 (0.63) | | -0.21 ± 0.16 | -1.35 (0.18) | -0.03 ± 0.13 | -0.21 (0.83) |
| Total sperm length (µm) | -0.06 ± 0.06 | -0.99 (0.32) | | 0.08 ± 0.06 | 1.36 (0.17) | -0.01 ± 0.05 | -0.29 (0.77) |
| + age | 0.06 ± 0.64 | 0.10 (0.92) | | 0.12 ± 0.67 | 0.17 (0.86) | 0.35 ± 0.59 | 0.60 (0.55) |
| + red border width | 0.07 ± 0.15 | 0.49 (0.62) | | -0.22 ± 0.16 | -1.39 (0.17) | -0.04 ± 0.13 | -0.33 (0.74) |
| Total sperm length (µm): quadratic | -0.002 ± 0.01 | -0.24 (0.81) | | 0.01 ± 0.01 | 1.33 (0.18) | 0.003 ± 0.004 | 0.85 (0.40) |
| F:H^b^ | -0.78 ± 0.71 | -1.09 (0.27) | | 0.29 ± 0.68 | 0.43 (0.67) | -0.59 ± 0.58 | -1.01 (0.32) |
| + age | 0.12 ± 0.65 | 0.18 (0.86) | | 0.23 ± 0.69 | 0.33 (0.74) | 0.45 ± 0.58 | 0.79 (0.43) |
| + red border width | 0.07 ± 0.14 | 0.51 (0.61) | | -0.20 ± 0.16 | -1.23 (0.22) | -0.02 ± 0.13 | -0.12 (0.91) |
| M:TSL^c^ | -2.59 ± 13.33 | -0.19 (0.85) | | -16.03 ± 14.21 | -1.13 (0.26) | -13.28 ± 11.76 | -1.13 (0.26) |
| + age | 0.07 ± 0.63 | 0.11 (0.91) | | 0.30 ± 0.74 | 0.41 (0.68) | 0.44 ± 0.57 | 0.77 (0.45) |
| + red border width | 0.06 ± 0.14 | 0.43 (0.67) | | -0.20 ± 0.17 | -1.21 (0.23) | -0.02 ± 0.13 | -0.14 (0.89) |
|  |  |  | |  |  |  |  |
| **Sperm motility** |  |  | |  |  |  |  |
| Velocity^d^ (µm/s) | 0.02 ± 0.01 | 1.27 (0.20) | | 0.001 ± 0.01 | 0.04 (0.97) | -0.01 ± 0.01 | -0.92 (0.45) |
| + age | -0.16 ± 0.75 | -0.21 (0.84) | | -0.27 ± 0.84 | -0.32 (0.75) | 1.11 ± 0.59 | 1.89 (0.09) |
| + red border width | 0.13 ± 0.18 | 0.73 (0.47) | | -0.47 ± 0.33 | -1.45 (0.15) | -0.09 ± 0.17 | -0.55 (0.58) |
| Proportion motile^e^ | -0.91 ± 1.74 | -0.52 (0.60) | | -1.73 ± 1.90 | -0.91 (0.36) | -2.02 ± 1.59 | -1.28 (0.21) |
| + age | 0.16 ± 0.70 | 0.23 (0.82) | | 0.20 ± 0.70 | 0.28 (0.78) | 0.54 ± 0.64 | 0.84 (0.41) |
| + red border width | 0.08 ± 0.16 | 0.50 (0.61) | | -0.21 ± 0.17 | -1.21 (0.23) | -0.10 ± 0.15 | -0.67 (0.51) |

^a^ Sperm morphology: *N* = 60/68/62
^b^ Flagellum to head ratio
^c^ Midpiece to total sperm length
^d^ Velocity: *N* = 50/57/52
^e^ Proportion motile: *N* = 56/64/58
